# Interaction of Somatic and Psychic Disorders

**Published:** 1897-03

**Authors:** Jas. G. Kiernan

**Affiliations:** Chicago, Ill.


### INTERACTION OF SOMATIC AND PSYCHIC DISORDER.

BY JAS. G. KIERNAN, M. D., CHICAGO, ILL.

The life of the poèt Keats contains so much illustrating the mental effects of phthisis that Rossetti, his biographer, is continually puzzled by them. The father of Keats was a stableman of bookish tastes. He seems to have been a natural-born gentleman, for even his marriage with his master's daughter (Keats' mother) left him unassuming and manly. Keats' mother was pleasure-seeking, yet saturnine. She displayed the same emotional mobility and suspicional tendency which characterized her gifted son. She died of phthisis complicated by rheumatism when he was fifteen years old. Keats, who was her first child, was born seven months after marriage. Haydon, the artist, states that Keats was, when a child, most violent and ungovernable. When five years old he once got hold of a naked sword and, shutting the door, swore that no one should go out. When his mother attempted to leave the room he threatened her so furiously that she began to cry and was obliged to wait till a man, who saw her through the window, came to her assistance. Keats during his childhood was irascible, suspicious, querulent and pugnacious. These mental states alternated with fits of emotional mobility and suspicional tenderness. During his mother's illness he nursed her with great care and tenderness.

To physicians the fact is of peculiar interest that Keats was apprenticed at the age of fifteen to a surgeon named Hammond. Five years later he passed his examination at Apothecaries' Hall, with great credit. In 1816 he became an interne at Guy's Hospital. In 1817 he contracted syphilis at Oxford, which was successfully treated by mercury. During the same year he was introduced to Coleridge, who prophesied his early death. In 1818 the first demonstrable symptom of phthisis became manifest. He had to leave Scotland and abandon his pedestrian tours, partly for this reason, partly because of the approaching death of his brother Tom, from phthisis. Tom, like Keats himself, had been irritable, querulent and suspicious as a youth. George, the other brother, who was pacific, albeit resolved, long survived Keats. The sister, who resembled George, died at an advanced age in 1890.

In September, 1818, Gifford's famous critique appeared in the *Quarterly*. Gifford was a fanatical philistinish devotee of the mechanical school of poetry founded by Pope. That "Endymion," the criticised poem, deserved a severely critical analysis is evident from Shelley's remark, "I have read Keats' poem. Much praise is due me for having read it, the author's intention appearing to be that no person should possibly get to the end of it." Gifford's savage onslaught was, therefore, somewhat merited, but it had altogether too much of the slapdash abusive style. The *Quarterly* critique had been preceded by a scurrilous attack on Keats in *Blackwood's*, by Lockard, Sir Walter Scott's son-in-law, which was revised by the latter. It was characterized by elephantinely jocose satire (?). The fact that Keats was "Johnny Keats", an apothecary's assistant, was the high and mighty reason assigned for shutting him out of Parnassus.

Shelley states, from hearsay, however, that Keats was driven by the *Quarterly* critique into a state bordering on insanity, which produced the first hæmoptysis. The first hæmoptysis, however, did not occur for more than a year after.

Haydon says: "The effect on Keats was melancholy. He became morbid and silent, would call and sit, whilst I was painting, for hours without speaking a word." Keats, however, had just such attacks of moody, suspicional taciturnity before.

In the preface to "Endymion", flayed so mercilessly by Gifford, Keats anticipates "a hell of criticism." This being the case, the mental effect could not have been so great. Moreover, the circumstances attendant on the first hæmoptysis were opposed to Shelley's opinion. Soon after the attack Keats is said to have been excessively addicted to claret and to laudanum. All were, however, so soon abandoned that they would appear to have been parts of some medical treatment tried by Keats.

The various brief attacks of irascibility described by his biographer from time to time, taken in conjunction with the physical symptoms, must convince any impartial mind of the justness of Dr. Richardson's view that Keats' death was due to phthisis attacking a frame predisposed to its ravages by inheritance. Keats' youngest brother, Tom, who bore such a decided mental and physical resemblance to him, died early of phthisis, January, 1820.

Keats became noted for his moody taciturnity, alternating with a suspicion which at times amountedalmost to panophobia, and became misanthropic, and the companionship of friends was tedious. He bewails from time to time his increasing lack of fixity of purpose, one of his earliest observed defects. The first hæmoptysis had been preceded by pulmonary symptoms dating from the visit to Scotland. It occurred a little over a year after the *Quarterly* critique, which, to judge from Keats' letters, does not seem to have left a very decided moral impression on him, since he did not exhibit more than the querulency and irritability common to one of his moods. A year later, however, near midnight one evening Keats, according to Lord Houghton, returned home in a state of "strange physical excitement," which to those who did not know him, might have appeared to be one of fierce intoxication. He had been chilled through on a stage coach, and soon after his return home spat up blood for the first time. His second attack occurred the next June, and thereafter Keats was so irritable, querulent and suspicious that the accidental opening of a letter by one of Leigh Hunt's children caused such impassioned outbursts that he left Leigh Hunt's house, despite all apologies and remonstranees.

The "fierce condition of physical excitement," which Lord Houghton likens to intoxication, and which seems to have been reported to Shelley as allied to insanity, was clearly an attack of emotional exaltation accompanied with irritability such as occur among the mental symptoms of phthisis. Rossetti is puzzled by the appearance of this "physical excitement resembling intoxication," at the time of the hæmoptysis, but such mental symptom is not infrequent precedent to hæmoptysis which it no doubt often provokes.

A singularly pat illustration of the emotional exaltation attendant on the first hæmoptysis is to be found in the frivolous, jaunty satire on George IV., written by Keats at this time, whose jocose tone under the circumstances puzzles Rossetti.

The following August after phthisis made its appearance, Keats, who had pronounced his own death-warrant on the first hæmoptysis, began to doubt his own diagnosis and believed his recovery certain.

His love-letters written precedent to the critique and for some time thereafter are, to quote Rossetti, unbalanced, wayward and profuse, full of suspicion from the outset, which is at first directed toward his friends and finally to his adored herself. Keats was well aware of this peculiarity of his, for he says: "You have all your life believed everybody; I have always suspected everybody."

September, 1820, Haydon found Keats, then on his way to Italy, helpless, despondent, irritable, suicidal. December, 1820, he was delirious part of the time, and had excessive longing for food. His mental condition varied. He was subject to unsystematized grandiose delusions, accompanied by suspicional irritability and followed by quiet stupor. He died February 23, 1821.

The prevalence of fashion in medical thought is illustrated by the fact that the Irish seventeenth century theory of the contagiousness of phthisis still prevailed at that time at Rome, and the room in which Keats died was treated as if an infected person had died therein.

Taking into account all the circumstances of Keats' career: His lack of fixity of purpose, which even Shelley found evident in "Endymion," his suspicional tendencies described by himself and others, the emotional mobility noted by Lord Houghton and others, and clearly evident in the jocose satire on George IV., his irascibility and pugnacity, as well as his heredity, the influence of phthisis on Keats' mental state is clearly manifest. If he had confined himself to the regular routine life of the medical practitioner, his life might have been preserved, since the emotional alternations of the life of the man of letters were certainly productive of vasomotor changes stimulating the mental and other symptoms of phthisis, and thus producing exhaustion.—*Alienizt and Neurologist.*

